# India ink artifact on ECG-gated SSFP sequences predicts resectability of tumours invading the mediastinum

**DOI:** 10.1186/1532-429X-14-S1-P53

**Published:** 2012-02-01

**Authors:** Lorenzo Monti, Maurizio V Infante, Tiziana Manias, Umberto Cariboni, Marco Alloisio, Luca Balzarini

**Affiliations:** 1 Cardiovascular Dept., I.R.C.C.S. Istituto Clinico Humanitas, Rozzano (MI), Italy; 2 Radiology, I.R.C.C.S. Istituto Clinico Humanitas, Rozzano, Italy; 3 Thoracic Surgery, I.R.C.C.S. Istituto Clinco Humanitas, Rozzano (MI), Italy

## Summary

Cardiac SSFP sequences can be used for the resectability assessment of advanced mediastinal tumors: the presence of India Ink artifact between tumor and structures predicts tumor resectability.

## Background

In tumours invading the mediastinum the identification of a resection plane between the mass and neighbouring critical structures may be doubtful on CT scans. Steady State Free Precession (SSFP) Magnetic Resonance (MR) sequences can show reciprocal sliding between structures, and a peculiar signal drop-out in voxels that contain both water and fat: the India Ink artifact. We tested if SSFP sequences can improve differentiation between contact and infiltration of mediastinal structures.

## Methods

Sites of contact between tumour and mediastinal structures on preoperative CT scans were individually assessed. 3 orthogonal stacks of SSFP sequences were acquired after contrast administration. Absence of infiltration was defined as the presence of India Ink artifact at the the tumour-structure interface. Surgical findings (presence or absence of a resection plane) were reported postoperatively for each site.

## Results

14 patients (9 males and 5 females, age 23-72) were preoperatively assessed by CT and MR. 6 patients had lung cancer, 6 thymoma, 1 sarcoma and 1 germ cell tumor. 69 contact sites were identified by preoperative CT scans. Surgical procedures were: pneumonectomy 5, mediastinal mass resection 5, extrapleural pneumonectomy 1, and exploratory thoracotomy 1. The negative predictive value was 0.94, positive predictive value 0.80, sensitivity 0.91, specificity 0.86, and accuracy 0.88. Complete resection was always successfully carried out based on MRI findings. In one case MR correctely anticipated non resectability.

## Conclusions

In patients with advanced mediastinal tumours and doubtful preoperative CT scan, SSFP-MR allows for an accurate selection and surgical planning of complex resections.

## Funding

None.

